# Patient Satisfaction and Clinical Efficacy of Novel Blood Glucose Meters Featuring Color Range Indicators in Patients With Type 2 Diabetes: A Prospective Study

**DOI:** 10.7759/cureus.11195

**Published:** 2020-10-27

**Authors:** Ayman Al Hayek, Asirvatham Alwin Robert, Mohamed Al Dawish

**Affiliations:** 1 Department of Endocrinology and Diabetes, Prince Sultan Military Medical City, Riyadh, SAU

**Keywords:** satisfaction, diabetes treatment, type 2 diabetes, contour® next one glucometer, clinical efficacy

## Abstract

Introduction

Self-monitoring of blood glucose (SMBG) plays an important role in diabetes management. The Contour^®^Next One glucometer is a recent glucometer that delivers blood glucose results by an immediate color indicator to aware users when blood glucose is at a critical high or low. The main purpose of the study was to assess the impact of an application of a blood glucose meter (BGM) having a color range indicator on clinical characteristics and glucose monitoring satisfaction (GMS) among patients having type 2 diabetes (T2D).

Methods

A total of 85 (male 42 and female 43) patients with T2D were switched to a BGM having smartLIGHT™ target range indicator (blood glucose meters featuring color range indicator) using Contour^®^Next One glucometer. Demographic data, as well as glycemic control, were collected at baseline and 12 weeks. At the time of the baseline and 12 weeks of the study, a trained interviewer gave the GMS survey questionnaire to every patient in order to collect the glucose monitoring satisfaction. In addition to GMS, a patient’s perceptions of smartLIGHT™ feature satisfaction survey responses were also collected from the patients at the end of the study (12 weeks).

Results

Compared to baseline, a significant improvement was observed in the subdomains of glucose monitoring satisfaction survey (GMSS) score on openness (p=0.0001), emotional burden (p=0.0001), behavioral burden (p=0.0001), and trust (p=0.0001) at the end of the study. Overall, the total GMS score at baseline was 2.61 ± 0.51, which improved up to 3.16 ± 0.63 (p=0.0001) during the period of 12 weeks. The patient satisfaction with smartLIGHT™ survey outcomes exposed evidence of satisfaction among the study population at the end of the study. There were statistically insignificant reductions observed in glycosylated hemoglobin (HbA1c) (p=0.063) and the frequency of hypoglycemia (p=0.057) at the end of the study.

Conclusions

The study demonstrates a significant improvement in the subdomains of GMSS - openness, emotional burden, behavioral burden, and trust - at 12 weeks than at the baseline, with the increased total GMSS score. Similarly, high satisfaction with the color-based smartLIGHT™ feature was also observed at the end of the study.

## Introduction

Diabetes is one of the major rising health threats of the 21st century. Over the past 20 years, the number of people living with diabetes has more than tripled, leading to life-threatening concerns if it is not managed properly [1, 2]. Over time, it can cause various macro- and microvascular complications, resulting in an overall reduction in blood flow [3, 4]. An increasing body of evidence suggesting that the structured advent of self-monitoring-of-blood-glucose (SMBG) helps all patients with type 2 diabetes (T2D) succeed in improvements in glycemic control, regardless of therapy. Notably, the Diabetes Control and Complications Trial confirmed that for patients who have diabetes, the SMBG is an essential factor in reaching good glycemic control and is related to the development of several complications of diabetes [5-8].

A number of hurdles to frequent SMBG have been recognized, and the necessity to be addressed in order to understand the full potential of SMBG in patients with T2D [9, 10]. Technologies for managing T2D continue to evolve, yet most patients with T2D have glycosylated hemoglobin (HbA1c) values above the target range [11, 12]. However, recent developments in glucose meters and SMBG data processing have revolutionized the clinical applicability of SMBG. These glucose meters deliver a fast analysis of blood glucose levels and permit the management of both hypoglycemic and hyperglycemic conditions to adjust glucose to a near-normal range, depending on the patient group [13].

To comfort the burden of self-care and consequently allow better glycemic control and satisfaction, people with T2D are turning to newer technologies for glucose monitoring such as Contour®Next One (Ascensia Diabetes Care, Parsippany, USA) utilizing color based smartLIGHT™ feature, which makes it quicker and easier to understand blood glucose readings using colored lights that identify if the reading is above, within or below the target range [6, 14, 15]. This is a highly accurate and easy-to-use glucometer, with its unique smartLIGHT™ feature (blood glucose meters featuring color range indicator), which instantly shows if blood glucose is in the target range, guiding decision-making support optimal diabetes self-management [6, 16]. The Contour®Next One blood glucose monitoring system (BGMS) also can be integrated with a smartphone app to simplify the management of diabetes. However, until now, not much research on the colored light range blood glucose meters (BGM) versus earlier model of glucometers in use. Hence, in the present study, we aimed to determine switching patients with diabetes to a novel BGM featuring a color range indicator (CRI) and its influence on clinical characteristics and glucose monitoring satisfaction (GMS) among T2D.

## Materials and methods

In this prospective study, we have carefully chosen a convenience sample of 85 adult patients with T2D (age range 30-70 years) who used their usual conventional BGM to self-test their glucose levels at least two times a day and patients treated from October 2019 to April 2020 at the Diabetes Treatment Center, Prince Sultan Military Medical City (PSMMC), Riyadh, Saudi Arabia. The sample size was calculated by Statulator, an online sample size calculator. Based on identifying a variance of one point in the test scores between baseline and three months (paired samples) with 95% confidence and 80% power, and consider a standard deviation of three in differences between baseline and three months, a minimum of 73 patients were required for this study. However, a final sample size of 85 patients was reached. 

Earlier to enrollment in the study, the participants had no experience using any CRI glucose meter. They were using their usual glucometer for SMBG at least two times per day for at least six months before enrollment. The study's exclusion criteria are: registering to another study, using another CRI glucose meter during the six months before inclusion in the study, and patients with type 1 diabetes. All participants were volunteers who own the right to withdraw from the research at any time, with or without any reason. All participants in the study received guidelines regarding their parts and signed informed consent before recruitment. The baseline visit was considered the first visit of the study. The following factors were documented on a standardized case record form: (1) demographic data and (2) clinical features. All patients were received a BGM featuring a target light indicator employing smartLIGHT™ feature using Contour®Next One glucometer at baseline, which also has an additional feature (second-chance) sampling that gives the patient a 60-second opportunity to apply more blood if the first sample was insufficient, which helps to avoid the need for repeat finger pricking. After 12 weeks, the clinical and satisfaction were collected using questionnaires. All processes followed were in accordance with the Helsinki Declaration of 1964, as revised in 2013. Approval of the study protocol was obtained from the Research and Ethics Committee of PSMMC, Riyadh, Saudi Arabia.

Glycosylated hemoglobin and hypoglycemia

An HbA1c level of < 7% reflected as good blood glucose level control. A confirmed blood glucose value of ≤ 70 mg/dL was defined as hypoglycemia. During the study, the HbA1c levels were analyzed twice, once at baseline and 12 weeks. The HbA1c levels were measured by the COBAS INTEGRA® 400 plus/800 analyzers (Roche Diagnostics, Indianapolis, USA) at the central laboratory of our hospital.

Glucose monitoring satisfaction survey

To evaluate the levels of glucose monitoring satisfaction survey (GMSS) subdomains such as openness, emotional burden, behavioral burden, and trust, we used the T2D version of the survey (GMSS version T2DM) [17, 18]. The GMSS survey version T2D includes a fifteen-item questionnaire, in which four items (questions number 1, 8, 10, and 14) belong to openness, another four items (questions number 2, 5, 9, and 13) belong to the subscale of emotional burden, four items (questions number 3, 6, 11, and 15) relate to the behavioral burden and three items (questions number 4, 7, and 12) fall in the category of trust. For rating the response to each item, a five-point Likert-type scale ranging from one (strongly disagree) to five (strongly agree) was employed where higher scores showed higher GMS [17, 18].

Survey of patient perceptions of smartLIGHT™ target glucose feature

Participant's smartLIGHT™ perception measures were assessed with a standard questionnaire (12 questions) at the end of the study, and they were asked to rate their experience with the system on a scale of one (strongly agree/painless) to five (strongly disagree/severe pain) [19].

Statistical analysis

Data analysis was carried out using Microsoft Excel 2010 (Microsoft Corporation, Seattle, USA) and the IBM SPSS Statistics for Windows, version 22 (IBM Corp., Armonk, USA). The continuous variables were represented as mean ± SD, while the categorical variables are shown as frequencies and percentages. The two-tailed paired t-test was executed to determine the differences between the different time points (baseline vs. 12 weeks). A p < 0.05 was considered to be statistically significant.

## Results

The clinical and demographic characteristics of the study population are shown in Tables 1-2. The study population's mean age was 46.7 ± 7.4 years, and 50.6% of the study sample were female. The majority of the study population was older than ≥45 years (62.4%), and 60% were diagnosed with diabetes for <10 years, and a total of 44.7% of participants had a body mass index 30-<30 kg/m^2^. The majority (65.8%) had high HbA1c values, and (77.6%) had a total daily insulin dose (TDD) of ≥0.7 Unit/Kg/Day.

**Table 1:** Baseline characteristics of the study population DM - diabetes mellitus

Variables	Frequency (n = 85)	%
Gender		
Male	42	49.4
Female	43	50.6
Age		
<45yrs.	32	37.6
≥45 yrs.	53	62.4
BMI		
25-29.9	21	24.7
30-<30 kg/m^2^	38	44.7
≥30 kg/m^2^	26	30.6
DM duration		
< 10 years	51	60
≥ 10 years	34	40

**Table 2:** Baseline clinical characteristics of the study population (n=85) HbA1c - glycosylated hemoglobin; TDD - daily insulin dose

Clinical characteristics	Frequency	%
Confirmed hypoglycemia episode/month		
1	5	8.3
2	14	23.3
3	21	35
4	14	23.3
>4	6	10
TDD/Unit/Kg/Day		
<0.7	19	22.4
≥0.7	66	77.6
HbA1c (%)		
<7	29	34.1
>7	56	65.8

**Figure 1:**
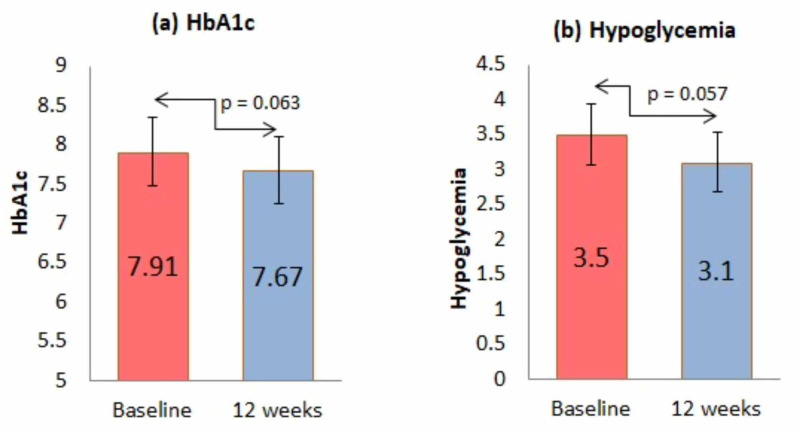
Influence of Contour® Next One glucometers on HbA1c and frequency of hypoglycemia HbA1c - glycosylated hemoglobin

Figure 1 showed the influence of Contour®Next One on HbA1c and the frequency of hypoglycemia. The baseline HbA1c level was 7.91% and 7.67% at 12 weeks (p=0.063). The baseline hypoglycemia frequency was 3.5, which decrease to 3.1 episodes/month (p=0.057).

**Figure 2:**
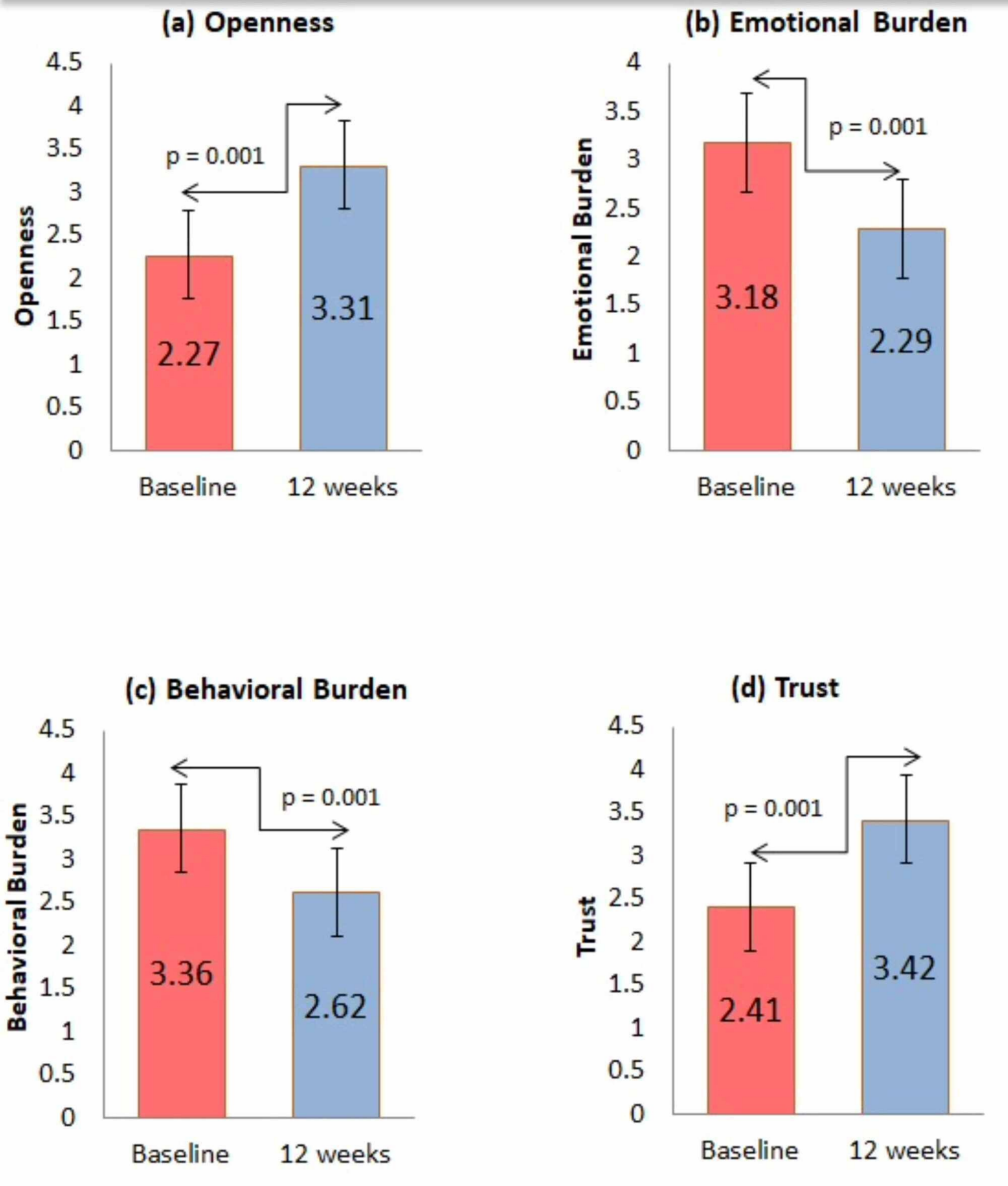
Influence of Contour®Next One glucometers on glucose monitoring satisfaction subdomains scores: (a) openness, (b) emotional burden, (c) behavioral burden, and (d) trust Total glucose monitoring satisfaction score baseline 2.61 ± 0.51, which increased up to 3.16 ± 0.63 (p=0.001).

Figure 2 compares the baseline and 12 weeks of GMSS subdomain scores among the studied population. The comparison points out a significant improvement in the subdomains of GMSS for openness (p=0.0001), emotional burden (p=0.0001), behavioral burden (p=0.0001), and trust (p=0.0001), at 12 weeks than at the baseline values, with the total GMSS score at baseline and 12 weeks being 2.61  ± 0.51 and 3.16 ± 0.63 (p=0.0001), correspondingly. During the follow-up, no episodes of severe hypoglycemia or serious device-related events happened.

The patient satisfaction with smartLIGHT™ survey results are presented in Table 3. Patient’s perceptions of contour smartLIGHT™ survey regarding the first statement show that 23.5% of the study population strongly agreed that the use of green for an in-range blood glucose result is intuitive and easy to understand. Similarly, 38.8% of the users strongly agreed that the smartLIGHT™ indicator is a helpful feature for identifying low and high blood glucose results, while 25.8% strongly agreed that the use of amber for a high blood glucose results is intuitive and easy to understand; 23.5% strongly agreed that the smartLIGHT™ feature provides visual assurance of the blood glucose result; 12.9% reported that they recommend this meter; 3.5% stated that the smartLIGHT™ feature helps them understand their blood glucose results; 23.5% felt that the using a meter with smartLIGHT™ feature set to their personal range helps them be more in control of their diabetes; 27% strongly agreed that the smartLIGHT™ feature is helpful for treating low blood glucose results; 20% reported that the smart smartLIGHT™ feature is helpful for treating high blood results; 29.4% reported that the use of red for a low blood glucose result is intuitive and easy to understand, and 41.1% participants strongly agreed that the smartLIGHT™ feature helps them to made correct treatment decisions.

**Table 3:** The responses of patient satisfaction with color based smartLIGHT™ feature survey statements

#	Questions	Strongly agree - 1	Agree - 2	Neither agree or disagree - 3	Disagree - 4	Strongly disagree - 5
1	The use of green for an in-range blood glucose result is intuitive and easy to understand	20 (23.5)	32 (37.6)	31 (36.4)	2 (2.4)	0
2	The smartLIGHT™ indicator is helpful for identifying low and high blood glucose results	33 (38.8)	37 (43.5)	15 (17.6)	0	0
3	The use of amber for a high blood glucose results is intuitive and easy to understand	22 (25.8)	32 (37.6)	29 (34.1)	1 (1.2)	1 (1.2)
4	The smartLIGHT™ feature provides visual assurance of my blood glucose result	20 (23.5)	35 (41.2)	21 (24.7)	7 (8.2)	2 (2.4)
5	I would recommend this meter	11 (12.9)	40 (47)	32 (37.6)	2 (2.4)	0
6	I would switch to a meter with smartLIGHT™ feature	20 (23.5)	44 (51.8)	21 (24.7)	0	0
7	The smartLIGHT™ feature helps me understand my blood glucose results	3 (3.5)	35 (41.2)	31 (36.4)	15 (17.6)	1 (1.2)
8	Using a meter with smartLIGHT™ feature set to my personal range helps me be more in control of my diabetes	20 (23.5)	31 (36.4)	32 (37.6)	2 (2.4)	0
9	The smartLIGHT™ feature is helpful for treating low blood glucose results	23 (27)	31 (36.4)	29 (34.1)	2 (2.4)	0
10	The smart smartLIGHT™ feature is helpful for treating high blood results	17 (20)	29 (34.1)	34 (40)	3 (3.6)	0
11	The use of red for a low blood glucose result is intuitive and easy to understand	25 (29.4)	27 (31.8)	25 (29.4)	8 (9.4)	0
12	The smartLIGHT™ feature helps me make correct treatment decisions	35 (41.1)	18 (21.2)	27 (31.8)	5 (5.9)	0

## Discussion

It is well recognized that regularly measuring HbA1c remains an essential tool for the treatment of diabetes [20]. However, HbA1c levels should not be the only conclusive factor for measuring the efficiency of diabetes treatment. Instead, patient-reported effects, comprising patient satisfaction, well-being, and quality of life, should also be given vital importance [21, 22]. Certainly, enhancement in treatment satisfaction may play a critical role in increasing patient self-efficacy and commitment to therapy, thereby supporting in reaching long-term stable glycemic control, in addition to the minimization of the risk of diabetic complications [18]. More recently, diabetes technology has extended, and studies reported that technological improvements in glucometers are enhanced diabetes management and patient satisfaction [19, 23-25]. In this present study, we explored whether the smartLIGHT™ based BGM can impact the clinical characteristics and GMS in T2D patients over a period of time.

After augmenting the blood glucose monitored by smartLIGHT™ based glucometer, in the present study, significant progress was detected in the tested factors of GMSS subdomains of openness, emotional burden, behavioral burden, and trust at 12 weeks as compared to baseline, along with the total GMS score being 2.61  ± 0.51 at baseline, which further improved up to 3.16 ± 0.63 (p=0.0001) at 12 weeks after the use of Contour®Next One featuring smartLIGHT™ technology. Previous studies also reported that CRI and wireless blood glucose meters increase patients' and healthcare professionals' satisfaction [26]. These new blood glucose meters not only lead to perfection in GMSS but also are connected with a significant improvement in diabetes management and metabolic control [24].

The patient satisfaction with smartLIGHT™ survey of the present study outcomes showed an evident of high-level satisfaction among the study population on the statements: intuitive and easy to understand, smartLIGHT™ indicator helps identify low and high blood glucose results, use of amber for a high blood glucose results is intuitive and easy to understand, smartLIGHT™ feature provides visual assurance of the blood glucose result, the feature helps to understand the blood glucose results, helpful for treating low blood glucose results and the use of red color for a low blood glucose result is intuitive and easy to understand. It is well demonstrated that diabetes technology has expanded. Studies reported that technological improvements in glucometers, including SmartLIGHT™ glucometers, improve diabetes management and patient satisfaction due to on-screen glucose range information using a color range indicator [6, 19, 23-25, 27]. Studies also reported that a blood glucose meter that provides automatic on-screen glucose range information using a color range indicator significantly improved the ability of patients having T2D to classify blood glucose readings into low range, in the range, or high glycemic range [6]. Also, other studies reported that switching patients to blood glucose meters with the feature of a color range indicator resulted in improvements in glycemic control compared to patients using currently marketed blood glucose meters that do not use a color range indicator [19, 27-29]. However, in our study, we found an improvement in HbA1c level and hypoglycemia frequency, but not statistically significant. This may occur due to the short duration of the study period and the limited number of patients tested. However, It is worthy to note that the frequency of self-testing among the studied population was 2.82 times/day at the baseline, while it was found to be higher in the smartLIGHT™ feature, i.e., 3.42 (difference of 0.6 times per day). The literature clearly demonstrated that the recommended SMBG frequency is useful for glycemic control. Moreover, a study from Germany also reported that the SMBG rate was significantly accompanying by good metabolic control with a decline of HbA1c of 0.20% for one additional SMBG per day [30].

Although the limitations that exist in the current research, such as (1) small sample size, (2) no randomization performed, no control group, and (3) the inclusion of a single-center for study, can be overcome by carrying out the study on a larger scale. With the above limitations, the present study brings important data about the smartLIGHT™ indicator feature-based blood glucose meter and delivers helpful insights regarding the significant positive improvement observed among adults with T2D due to replacing the previous usual method with the smartLIGHT™ blood glucose meter. 

## Conclusions

Conclusively, this prospective study's outcomes clearly demonstrated that using the color-based smartLIGHT™ blood glucose meter can upsurge the GMS. On the other hand, no statistical differences were detected on the HbA1c level and hypoglycemia. It may occur due to the short duration of the study period. However, further studies are mandatory for ascertaining whether the continued and consistent use of the color based blood glucose meter will result in improved outcomes on GMSS.
